# Amelioration of Sickle Cell Pain after Parathyroidectomy in Two Patients with Concurrent Hyperparathyroidism: An Interesting Finding

**DOI:** 10.1155/2016/3263951

**Published:** 2016-08-04

**Authors:** John Muthu, Mir Ali

**Affiliations:** ^1^Sickle Cell Division, Department of Medicine, New York City Health and Hospitals/Kings County, Brooklyn, NY 11203, USA; ^2^Interfaith Medical Center, Brooklyn, NY 11213, USA

## Abstract

Patients with sickle cell disease have high morbidity and healthcare utilization due to repeated painful crises. Some coexisting conditions which cause pain similar to sickle cell disease may go undiagnosed in these patients. We report two adults with concurrent hyperparathyroidism who experienced significant improvement in sickle cell pain following parathyroidectomy thereby pointing to hyperparathyroidism as the principal causative factor for their pain. Meticulous evaluation for parathyroid disorders can be rewarding in sickle cell disease.

## 1. Introduction

Sickle cell disease (SCD) is a genetic disorder which affects approximately 100,000 Americans [1]. This disease is characterized by production of an abnormal hemoglobin (Hb) molecule which polymerizes into long chains when deoxygenated. This leads to deformation of red blood cells (RBCs) into their characteristic sickle shape. Vasoocclusion by sickle shaped RBCs and their hemolysis are the hallmarks of SCD. Acute and chronic body pains which are common clinical symptoms in SCD are understood to be due to vasoocclusion [2]. All painful events in SCD may not be sickle cell related. Other systemic disorders including some endocrine disorders like hyperparathyroidism which commonly cause musculoskeletal pain may present concurrently with SCD. Here we report two patients with concurrently occurring hyperparathyroidism who experienced amelioration of sickle cell pain following parathyroidectomy. There is a prior case report of primary hyperparathyroidism mimicking vasoocclusive crises in a pediatric patient with homozygous sickle cell (HBSS) disease [3]. Our case report exemplifies that the benefit of decrease in painful episodes following treatment of hyperparathyroidism may be seen in other genotypes like sickle cell-beta^+^ thalassemia. We also report a similar benefit following treatment of tertiary hyperparathyroidism due to end stage renal disease occurring concurrently in a patient with HBSS disease.

## 2. Case Presentation 

Baseline characteristics of the two patients at the time of diagnosis of hyperparathyroidism are summarized in Table 1.

### 2.1. Case Report 1

Our first case is a 59-year-old female patient who moved to USA from Haiti two years ago with a diagnosis of sickle cell-beta^+^ thalassemia. She reported having generalized pain almost on a daily basis which she managed at home with pain medications. She had frequent hospital visits for generalized painful crises as many as twice a month of which some required inpatient treatment. We analyzed her case in detail after one such prolonged admission for 15 days especially as the severity of her presentations for painful crises was unusual given her sickle cell genotype.

High Performance Liquid Chromatography (HPLC) showed HbA of 22.4%, HbA2 of 7%, HbS of 65.4%, and HbF of 5.2% consistent with a diagnosis of sickle cell-beta^+^ thalassemia. Hemogram showed white blood cell (WBC) count of 4.3, Hb of 9.8 grams/deciliter (g/dL), and platelet (Plt) count of 158. Blood chemistry was significant for elevated calcium (Ca) on multiple occasions with a peak of 12.7 milligrams/deciliter (mg/dL). Phosphate was low on multiple occasions with a nadir of 1.9 mg/dL. Parathyroid hormone (PTH) level was elevated on multiple occasions with a peak of 147 picograms/milliliter (range 14–72 pg/mL). Vitamin D levels ranged from 18 to 23 nanograms/milliliter (range 30–100 ng/mL). Based on the above investigations we reached a diagnosis of primary hyperparathyroidism (PHPT).

Patient underwent imaging studies to localize the parathyroid lesion. Radionuclide parathyroid imaging using technetium sestamibi scan revealed slow washout in the right mid thyroid region (Figure 3) and computed tomography (CT) scan of the neck without contrast revealed a soft tissue mass posterior to mid pole of the right thyroid lobe (Figure 4) both favoring a diagnosis of adenoma of the right inferior parathyroid gland. She also had intermittent upper abdominal pain which was confirmed on upper gastrointestinal endoscopy to be due to severe gastritis.

**Figure 1:**
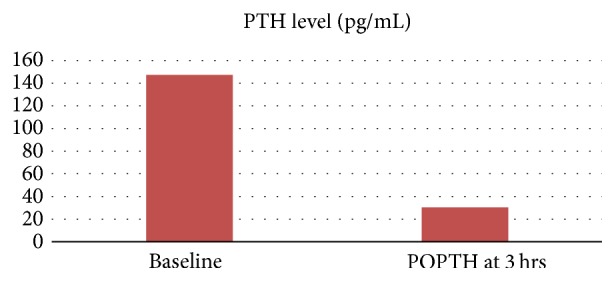
Perioperative PTH monitoring, POPTH (postoperative PTH), and Pg/mL (picograms/milliliter).

**Figure 2:**
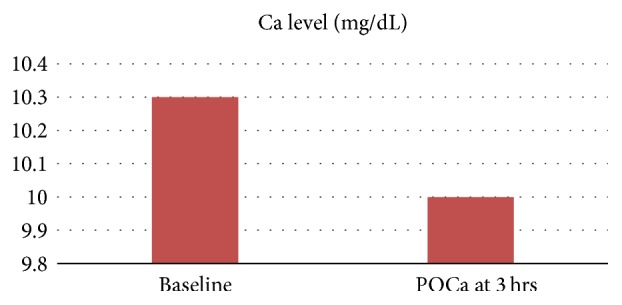
Perioperative calcium monitoring, POCa (postoperative calcium), and Mg/dL (milligrams/deciliter).

**Figure 3:**
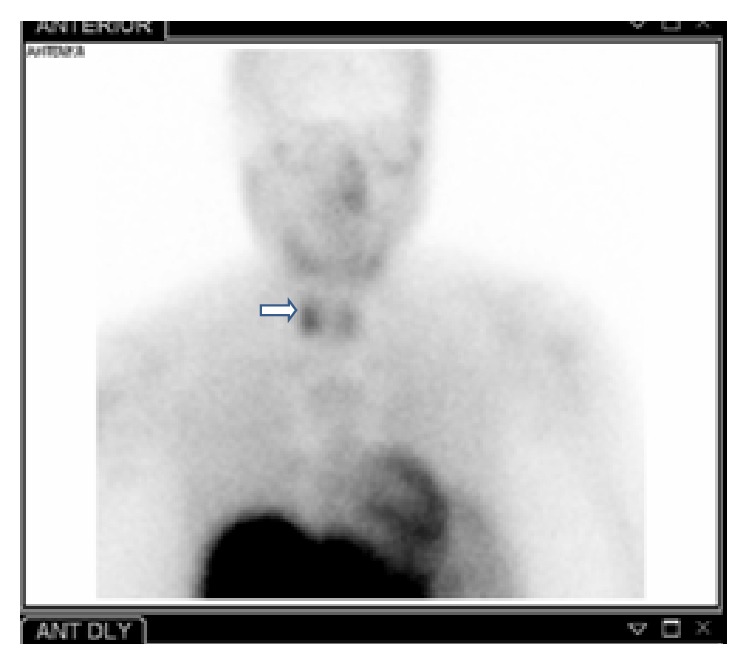
Radionuclide parathyroid imaging using technetium Tc-99m sestamibi showingslow washout in the right mid thyroid region (denoted by arrow).

**Figure 4:**
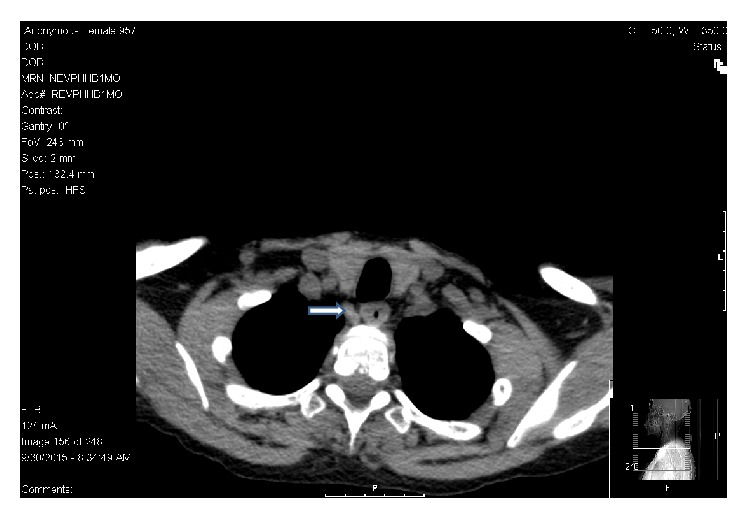
CT scan of the neck without contrast showing a soft tissue mass posterior to mid pole of the right thyroid lobe (denoted by arrow).

Patient underwent focused parathyroidectomy of the right inferior parathyroid gland which was confirmed on histopathologic examination to be a parathyroid adenoma. Intraoperative PTH monitoring was not used in this case. Patient's calcium and PTH levels normalized following surgery and are shown in Figures 1 and 2. At 6-month follow-up calcium level was 9.7 mg/dL and PTH was 28.4 pg/mL confirming cure of PHPT. Following successful treatment of PHPT, she has not had any sickle cell related painful events and has not required emergency room (ER) visits or hospitalizations over the last year.

### 2.2. Case Report 2

Our second case is a 26-year-old male patient with homozygous sickle cell (HBSS) disease. He also had chronic kidney disease which progressed over 3 to 4 years to end stage renal disease (ESRD) requiring hemodialysis. Patient had frequent presentations for painful crises amounting to 16 ER visits and 82 inpatient days in the preceding year at our institution. His painful crises were notably more frequent since he developed ESRD and were localized in his hips and collar bones.

Most recent HPLC showed HbA of 81.8%, HbF < 1%, HbA2 of 2.7%, and HbS of 14.5% indicating that patient had recently received multiple blood transfusions. Hemogram showed WBC count of 12, Hb of 7.1 g/dL, and platelet count of 333. Blood chemistry was significant for elevated Ca on multiple occasions with a peak of 11.8 mg/dL. Phosphate was high on multiple occasions with a peak of 9.1 mg/dL. PTH was elevated on multiple occasions with a peak of 4078 pg/mL. Vitamin D levels ranged from 13 to 20 ng/mL. Predialysis blood urea nitrogen ranged from 60 to 80 mg/dL and creatinine ranged from 8 to 10 mg/dL. Patient was treated with cinacalcet for secondary hyperparathyroidism but became refractory to treatment after a period of time with persistently elevated calcium and PTH levels. Based on the above investigations, we reached a diagnosis of tertiary hyperparathyroidism due to ESRD.

Imaging studies were done to localize the parathyroid lesion. Radionuclide parathyroid imaging using technetium sestamibi scan showed slow washout in the right lower thyroid region (Figure 7) but CT scan of the neck with contrast showed moderately enhancing foci inferior to the left and right thyroid lobes and superior to the left thyroid lobe which raised suspicion of parathyroid hyperplasia and warranted exploration of all four parathyroid glands. Plain radiography revealed bony resorption typical of untreated hyperparathyroid bone disease at the greater trochanters of both femoral bones and lateral ends of both clavicles which were the locations of his pain (Figures 8 and 9).

**Figure 5:**
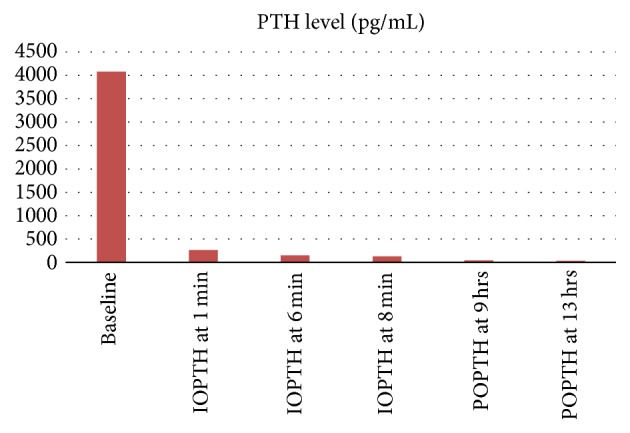
Perioperative PTH monitoring, IOPTH (intraoperative PTH), POPTH (postoperative PTH), and Pg/mL (picograms/milliliter).

**Figure 6:**
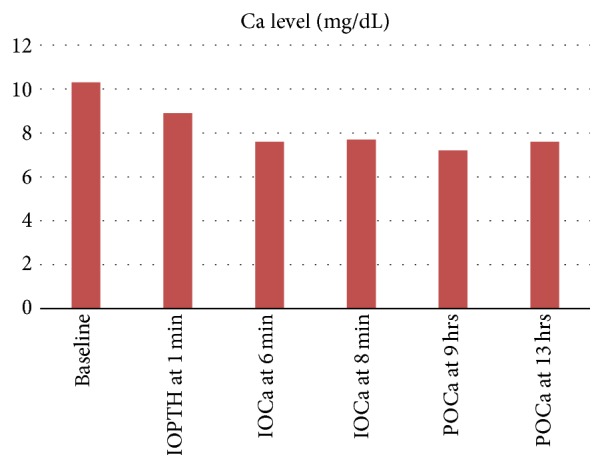
Perioperative calcium monitoring, IOCa (intraoperative calcium), POCa (postoperative calcium), and Mg/dL (milligrams/deciliter).

**Figure 7:**
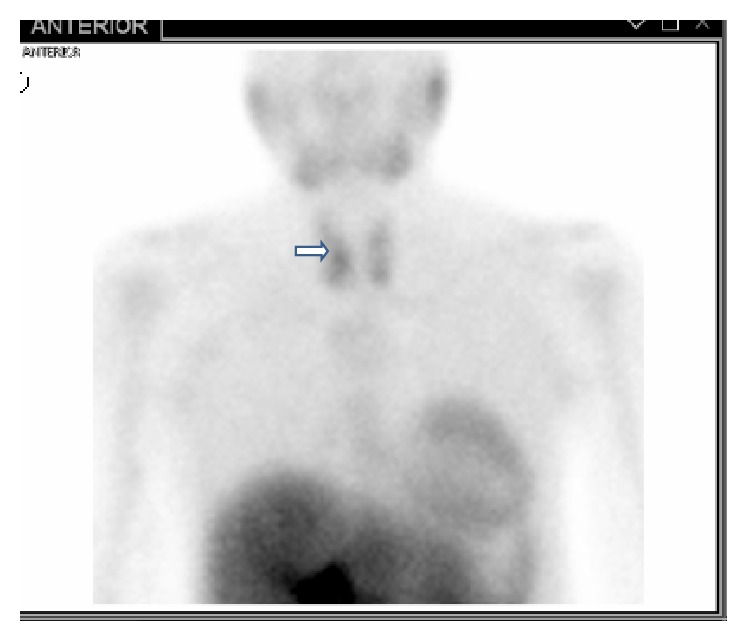
Radionuclide parathyroid imaging using technetium Tc-99m sestamibi showingslow washout in the right lower thyroid region (denoted by arrow).

Four-gland exploration and parathyroidectomy were performed and a portion of one gland was reimplanted in the left sternohyoid region. Histopathology showed parathyroid hyperplasia of all the resected glands. Intraoperative PTH monitoring was used as shown in Figures 5 and 6. At 6-month follow-up calcium level was 9.1. Since this patient underwent parathyroidectomy, his frequency and severity of painful crises have decreased significantly. He has had only 3 ER visits and a total of 8 inpatient days for painful crises in the year following parathyroidectomy which is significantly lower than before the surgery.

## 3. Discussion 

Vasoocclusive episodes in SCD involve multiple organs with musculoskeletal system being most commonly affected. Some patients have acute generalized body pain while others have localized bony pain resulting from bone infarcts [4]. The long bones are most commonly affected followed by ribs, sternum, and vertebral bodies. The pain may range from mild to very severe. Many individuals with SCD also suffer from long term consequences of vasoocclusive pain episodes in the musculoskeletal system, such as avascular necrosis of the femoral heads or collapsed vertebral bodies. This leads to a chronic state of pain in addition to the more acute painful episodes. Pain symptoms are a major cause of morbidity and affect quality of life in these patients. These symptoms result in multiple ER visits and hospitalizations. They are managed with pain medications, intravenous fluids, and blood transfusions as needed along with treatment of the underlying trigger. SCD may be associated with endocrine disorders like hyperparathyroidism which also cause generalized body pains. It is important to identify and treat these disorders for an optimal outcome and not to dismiss all pain symptoms in these patients as being due to SCD.

Hyperparathyroidism may be primary, secondary, or tertiary. Primary hyperparathyroidism (PHPT) is due to oversecretion of PTH most commonly from a parathyroid adenoma [5]. Most new patients are asymptomatic at the time of diagnosis with PHPT. Biochemical tests that include measurement of serum calcium currently account for identification of at least 80 percent of patients with PHPT in western countries [6]. The classical symptoms of PHPT are due to combined effects of increased PTH and calcium. Complaints of weakness and fatigue are common among patients with PHPT [7]. Other manifestations include nephrolithiasis, bone disease, constipation, polyuria, and polydipsia. There is a wide spectrum of involvement of the skeletal system in hyperparathyroidism. Effects can range from generalized bone pains to asymptomatic patients with decreased bone densitometry and increased risk of fractures [8]. Secondary and tertiary hyperparathyroidism occur in patients with chronic kidney disease. Tertiary hyperparathyroidism is characterized by severe parathyroid hyperplasia with autonomous secretion of PTH that is no longer adequately responsive to the plasma calcium concentration. This causes high bone turnover and abnormal mineralization [9]. Musculoskeletal symptoms from hyperparathyroidism can be difficult to distinguish from those in patients with concurrent SCD.

There are variations in clinical presentation among the different sickle cell genotypes with homozygous HBSS disease and heterozygous sickle cell-beta^0^ thalassemia patients being the more severely affected ones. In HBSS disease fetal hemoglobin (HbF) is a major modulator of polymerization in that the higher the HbF levels, the more benign the clinical and hematologic features of sickle cell anemia [10].

Some heterozygous sickle cell variants like sickle cell-beta^+^ thalassemia and HBSC disease generally have a more benign clinical course. In sickle cell-beta^+^ thalassemia, normal adult hemaoglobin (HbA) accounts for 18 to 25 percent of total hemoglobin which prevents extensive polymerization. Similarly, in HBSC disease, HBC hemoglobin does not participate in polymerization. Therefore, in both these variants painful events occur at less than half the freuency as the HBSS type [11, 12]. Our first patient with sickle cell-beta^+^ thalassemia was having more frequent painful episodes than expected for her genotype. This prompted us to investigate for other coexisting illnesses and we reached a diagnosis of primary hyperparathyroidism. The marked improvement in her pain symptoms following resection of the parathyroid adenoma made it clear that hyperparathyroidism had been partially responsible for the pain symptoms.

**Figure 8:**
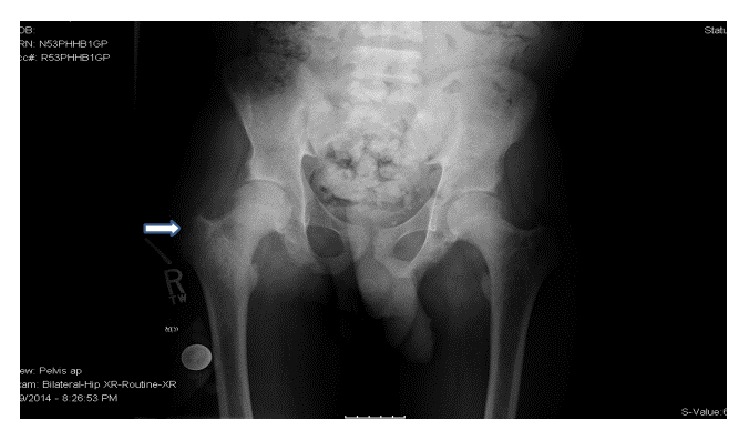
Bony resorption of greater trochanters of both femoral bones typical of untreated hyperparathyroid bone disease at the locations of the patient's pain (denoted by arrow).

**Figure 9:**
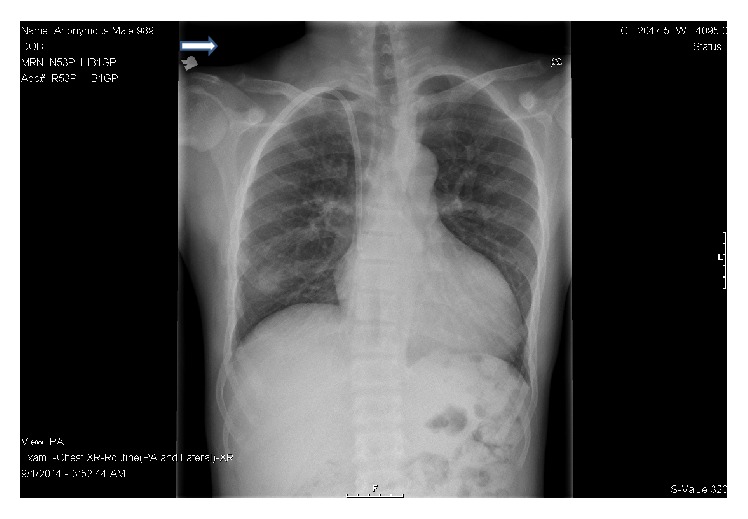
Bone resorption of the lateral ends of both clavicles (denoted by arrow).

Our second patient with HBSS disease and ESRD received multiple transfusions for symptomatic anemia not responding to erythropoietin. Though his HbA was >80%, he did not have the benign course in terms of painful events which would be expected with low HbS levels of 14.5%. This led us to evaluate for other coexisting pathologies for his musculoskeletal pain. There were radiological features of untreated hyperparathyroid bone disease at the locations of his pain. There was bone resorption of greater trochanters of both femoral bones and lateral ends of the clavicles but there were no bone infarcts or avascular necrosis of the femoral heads as would be anticipated in SCD (Figures 8 and 9). These radiological findings along with analysis of the case as described above led to our conclusion that hyperparathyroidism was more likely than SCD to be the cause of his frequent painful episodes.

**Figure 10:**
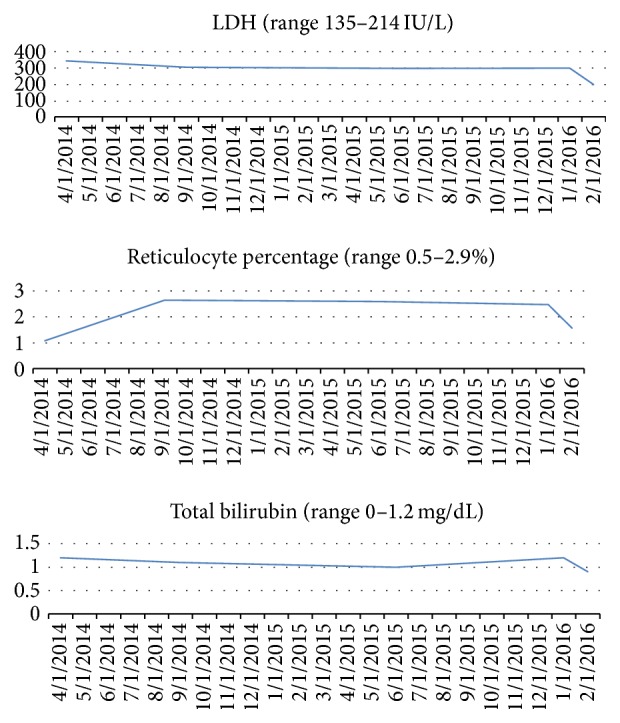
Graphs showing lactate dehydrogenase (LDH), reticulocyte percentage, and total bilirubin levels in the first patient.

Patients with phenotypic manifestations which are incongruous with their genotype, that is, more severe and frequent pain symptoms in a milder sickle cell variant, should be investigated for concurrent systemic disorders which can contribute to their pain. Similarly frequent or persistent painful episodes despite relatively normal markers of active sickle disease like lactate dehydrogenase (LDH), reticulocyte count, and bilirubin should also prompt further investigations. Notable findings in our first patient include LDH, reticulocyte percentage, and bilirubin levels which stayed at baseline during painful events over a 22-month period which is unlike that seen in sickle cell crises (Figure 10).

The improvement in sickle cell pain in our two patients following parathyroidectomy points towards hyperparathyroidism being the predominant cause of their pain. Further studies need to be done to see if there is a potential causal relationship between the two conditions. There are other potential benefits of treatment of hyperparathyroidism in patients with SCD. Patients with SCD have a high rate of vitamin D deficiency and osteoporosis [13]. Concurrent hyperparathyroidism causing a high bone turnover may worsen osteoporosis in patients with SCD. Treatment of hyperparathyroidism in appropriate cases with parathyroidectomy will help prevent long term bone damage. In addition it is expected that there will be an improvement in muscle strength following parathyroidectomy [14]. There may also be an added benefit in preventing opioid dependence in this subset of patients whose pain is commonly attributed to sickle cell disease.

## 4. Conclusion

Hyperparathyroidism can occur concurrently with sickle cell disease and can cause symptoms which mimic sickle cell painful events. Timely diagnosis and treatment of hyperparathyroidism can have beneficial effects in patients with sickle cell disease.

## Figures and Tables

**Table 1:** Hb: hemoglobin, HPLC: high performance liquid chromatography, HbS: sickle cell hemoglobin, HbF: fetal hemoglobin, HbA and HbA2: adult hemoglobin, and PTH: parathyroid hormone.

	Case 1	Case 2
Age	59	26
Gender	Female	Male
Sickle cell genotype	Sickle cell-beta^+^ thalassemia	Homozygous sickle cell (HBSS)
Hb (range 14–18 mg/dL)	9.8	7.1
Reticulocyte (range 0.5–2.9%)	2	5
Lactate dehydrogenase (range 135–225 U/L)	300	269
Total bilirubin (range 0.0–1.2 mg/dL)	1.2	0.7
HPLC		
HbS%	65.4	14.5
HbF%	5.2	<1
HbA%	22.4	81.8
HbA2%	7	2.7
Calcium level (range 8.4–10.3 mg/dL)	12.7	11.8
Phosphate level (range 2.7–4.5 mg/dL)	1.9	9.1
PTH level (range 14–72 pg/mL)	147	4078
Vitamin D level (range 30–100 ng/mL)	23	20
Pathologic diagnosis	Parathyroid adenoma	Parathyroid hyperplasia
